# Effect of COVID-19 on Geriatric Medical Services in China

**DOI:** 10.14336/AD.2021.0703

**Published:** 2022-02-01

**Authors:** Qingqing Yin, Xueying Liu, Chengcheng Huang, Wenkai Bi, Runfa Zhou, Renjun Lv

**Affiliations:** ^1^Department of Geriatric Neurology, Shandong Provincial Hospital Affiliated to Shandong First Medical University, Jinan, Shandong, China.; ^2^Department of Endocrinology, Shandong Provincial Hospital Affiliated to Shandong First Medical University, Jinan, Shandong, China.; ^3^Clinical Education Administration, Affiliated Hospital of Shandong University of traditional Chinese Medicine, Jinan, China.; ^4^Department of Nuclear Medicine, Shandong Provincial Hospital Affiliated to Shandong First Medical University, Jinan, Shandong, China.; ^5^Department of Cardiology, Shandong Provincial Hospital Affiliated to Shandong First Medical University, Jinan, Shandong, China.; ^6^Department of Respiratory Medicine, the First Hospital of Lanzhou University, Lanzhou, China.

## To the Editor,

At present, in China there are more than 250 million people over the age of 60 years, 75% of older people are diagnosed with more than one chronic disease, and approximately 40 million have disabilities or experience partial disabilities. These problems not only seriously influence the quality of life of older people, but also place a heavy burden on families and society. According to the Plan of Health China 2030 (www.gov.cn/xinwen/2016-10/25/content_5124174.htm), older people should be provided with health care and geriatric services that include hospitalisation during treatment periods, nursing during rehabilitation, daily care during the stable periods, and hospice care. Currently, the development of appropriate geriatric medical services in China has fallen behind, and there are several shortcomings. Many hospitals still focus on specialist care rather than treating older people as a population. With the development of modern medicine, hospital divisions have become increasingly specialised, for instance, the cardiology unit only treats heart disease, the respiratory unit only treats respiratory disease, and the orthopaedic unit only treats orthopaedic disease. If an older person is diagnosed with multiple comorbidities, the patient will often need to be rushed to different specialised hospitals, register with different doctors, and will ultimately be prescribed several medicines and treatments. Thus, a medical model that focuses on diseases or organs rather than patients does not easily adapt to the changing needs of an ageing society. Additionally, there is a lack of communication between general care hospitals and community hospitals, and older individuals are unable to receive continuous treatment and care in the community following discharge from general hospitals.

Since the initial reports of Coronavirus Disease 2019 (COVID-19) in December 2019, pneumonia caused by severe acute respiratory syndrome coronavirus 2 (SARS-CoV-2) has spread rapidly worldwide, causing a global pandemic. The COVID-19 epidemic has brought new challenges to geriatric medical services and has also promoted the improvement of geriatric medical services in China. This paper analyses the impact of the COVID-19 epidemic on geriatric medical care, the prevention and control approaches that can be established, and the challenges these preventative and control measures present for geriatric medical services in China in the future.

## Establishment of an independent infectious disease prevention and control infrastructures in geriatric care hospitals

The requirements for the prevention and control of respiratory infectious diseases are not clear in the guidelines regulating the construction of geriatric hospitals. For example, do geriatric hospitals need to set up fever clinics? Is physical isolation from the primary hospital facility necessary? What are the volume and size requirements of a negative pressure isolation ward? Considering the characteristics of the COVID-19 outbreak, these aspects are worthy of attention in geriatric specialty hospitals. Thus, the government should seriously address the needs for prevention and treatment of infectious diseases, infrastructure construction, and subsequent operation policies once the hospital is established. For example, fever clinics, isolation areas, and dedicated areas for health assessment, physical examinations, and rehabilitation for older patients should be located on the first floor of the building; epidemic prevention areas should be independent and distanced from medical areas; and normal medical areas should be limited to the greatest extent possible.

Comprehensive consideration should be given to severe infectious diseases and the treatment and health care of older individuals. The "seven stages" management of geriatric diseases includes health promotion, prevention and health care, prevention and control of chronic diseases, acute medical care, mid-term care, long-term care, and hospice care. Considering the COVID-19 epidemic, it can be concluded that geriatric hospitals need "an independent infectious disease prevention and control program", which includes an independent fever clinic, an independent isolation ward, and an independent hospital infection management strategy, which will ensure the provision of continuous medical services for older individuals and will allow to gradually develop a friendly culture, management, services, environment, and a relatively safe medical setting.

## Improve the community health system network

Historical experience on the prevention and control of major infectious diseases has confirmed that the community health system is very important [1-3]. Grassroots health systems and community physicians are the first line of defence in the prevention and control of transmission of infectious diseases [4] and are also the most effective means of defence against external input and internal containment [5]. Community health policies have played a significant role in the construction of medical services for older people, which not only identifies the problems and difficulties faced in seeking medical care by community-dwelling people but also helps community-dwelling individuals to reduce the costs of seeing doctors. In addition, the pressure of hospital diagnosis and treatment during the epidemic has been alleviated.

The COVID-19 epidemic has exposed the underlying problems in China's current medical and community health system, including inadequate capacities for essential health services, lack of experience in prevention and control of infectious diseases, and the promotion of graded diagnosis and treatment protocols still need to be further strengthened. Most of China’s medical and health resources are concentrated in large general hospitals; thus, grassroots medical and health institutions have limited medical resources, lack professional team support, and the capacity of fundamental medical and health services is not high; thus, it is difficult for these institutions to play a role in disease prevention and control. Considering lessons learned from previous practical experience [1], community medical staff, the information system, and procedures can be improved and updated by strengthening institutional training, professional skills training, and continuing education [6]. Acceleration of the construction of a health information network for older adults is necessary. Currently, medical records for individual patients are scattered; they are only available internally to community health service organisations and are not shared with specialist hospitals. It is therefore necessary to establish a unified network of medical health records and information systems for older patients as soon as possible to achieve a more integrated management of community health institutions and specialist hospitals, to allow medical institutions at all levels a timely and rapid understanding of the basic health status of older patients and hence, provide more effective services.

## Upgrade and standardisation of the management of nursing homes

At the end of December 2020, there were 38,000 retirement institutions in China (www.mca.gov.cn/article/xw/mzyw/202102/20210200032002.shtml).

Nursing homes (NHs) are a congregate living setting, most NHs have rooms housing more than one occupant with shared bathrooms, which facilitates the transmission of the virus. Furthermore, most senior citizens have multiple comorbidities and/or present an immunocompromised state that predispose them to acquiring SARS-CoV-2, or more atypical presentations of COVID-19. NHs are considered hotspots for COVID-19, given the residential environment and patient vulnerabilities, and as the COVID-19 pandemic continues to unfold, several challenges will affect NHs. At present, the traditional Chinese pension model mainly provides life care services, although to a limited degree. In the face of a sudden epidemic crisis, these services are unable to adequately address unexpected nursing responsibilities. However, due to the small number of NHs combining medical care and nursing care, the shortage of professional nurses, the limited number of services offered, and inadequate nursing education specific to older individuals, the training of healthcare personnel in our country is still at the beginning [7]. The level of nursing staff is uneven, there is a lack of systematic and standardised essential knowledge and professional skills training, and most nursing is limited to basic life care, but lacks human-centred care. This requires us to integrate geriatric care, medical care, rehabilitation care, and hospice care, to ensure a balanced combination of old-age care and medical resources, and thereby upgrade the traditional approach of providing for the aged.

A clinical unit should be set up in institutions for the aged. Administrative offices, similar to those of basic hospitals, should also be established in NHs. This model requires the selection of retirement facilities with suitable infrastructure and beds, which will provide comprehensive services for the elderly, such as everyday necessities, medical care, nursing and psychology services. Retirement institutions and medical institutions form a bi-directional collaborative system. The hospital provides basic nursing training for nursing staff in NHs and carries out regular health education sessions. In addition, when the residents become sick, they are immediately sent to a nearby collaborative hospital for treatment. After the condition is improved and stable, they can return to the NHs for follow-up rehabilitation physiotherapy. Training professional talents and improve service quality. Strengthening training in geriatric care, rehabilitation, nursing, and other professional skills, improves an individual’s culture baggage and professional level, expands the application of appropriate geriatric health care, health care training programs, and require all regions to provide organised training.

In the face of any potential future public crisis situations, NHs should establish crisis prevention and control protocols (www.mca.gov.cn/article/xw/tzgg/202002/20200200024221.shtml). The director or person in charge of the retirement institution is fully responsible for any preventive measures and controls—they establish and implement the prevention and control guidelines and the emergency plan; assign the available areas in the facilities; and are responsible for establishing a systematic process of nosocomial infection control, isolation protocols, and organising their implementation. A 24-hour emergency watch system should be established to ensure smooth communication, timely report of information as necessary, no delayed reports, concealments, or omission. Through announcements, phone calls, text messages (via WeChat or e-mail for example) and other means should be provided to the elderly and their families by the retirement institutions regarding the epidemic prevention and control procedures and any related service notices.

## Strengthening of homecare medical services for older individuals

In the early stages of the epidemic, communities and institutions implemented a lockdown management approach; thus, it became inconvenient for older patients requiring medical treatment or medicine to leave and return to the institution, and patients often exiting the institution for medical treatment would also face a greater risk of infection. In addition, older or disabled older patients with chronic diseases, recovering from illness or in terminal stages, and older patients who require medical services after discharge have a strong demand for home medical services [8]. Homecare services involve medical staff from institutions providing door-to-door medical care such as diagnosis and treatment services, medical care, rehabilitation treatment, pharmaceutical care, hospice care, and traditional Chinese medicine services for older patients according to the individual’s specific requirements. Medical systems (essential health services) can provide medical services at home by providing beds, door-to-door visits, and contracts by family doctors. This system will allow not only to optimise the rational use of community health resources and will reduce the medical burden of older patients but will also provide a more suitable medical setting for care of older persons, avoiding the fatigue associated with travelling to and from the hospital, and allow patients to receive treatment in a comfortable family environment. Furthermore, this approach has a positive soothing effect on the psychological welfare of the older patient. Through the medical institutions, "Internet + Medical Health" services, telemedicine, the medical system can be extended to the patient’s home and will contribute to establishing innovative home-based medical services (www.nhc.gov.cn/yzygj/s7653pd/202012/19a2617ba8e641bea9ac2472ea04c82a.shtml).

Based on the current situation in China, medical services for older patients has adopted a model of considering the older person’s family as the foundation, the community as the centre, and the hospital as the support, allowing patients to take full advantage of community health services, and making the provision of continuous and comprehensive medical services to older patients the focus of medical and health services. Given the common medical needs of older patients, policy makers should take the initiative of visiting individuals door-to-door to further improve integration within the community. This would ensure that older individuals have access to efficient medical services while maintain the existing physical well-being of older patients and improving their health status and quality of life to the greatest extent possible. The establishment of a medical service system guiding the collaboration between hospitals-community health service centres (stations)-general practitioners, nurses-caregivers, will form a cohesive strategy encouraging close cooperation, to create an innovative medical service model able to satisfy the needs of older individuals.
